# Dysregulated Alanine as a Potential Predictive Marker of Glioma—An Insight from Untargeted HRMAS-NMR and Machine Learning Data

**DOI:** 10.3390/metabo11080507

**Published:** 2021-08-01

**Authors:** Safia Firdous, Rizwan Abid, Zubair Nawaz, Faisal Bukhari, Ammar Anwer, Leo L. Cheng, Saima Sadaf

**Affiliations:** 1 School of Biochemistry and Biotechnology, University of the Punjab, Lahore 54590, Pakistan; safia.phd.ibb@pu.edu.pk (S.F.); rizwan.phd.ibb@pu.edu.pk (R.A.); 2 Riphah College of Rehabilitation and Allied Health Sciences, Riphah International University, Lahore 54770, Pakistan; 3 Department of Data Science, Punjab University College of Information Technology, University of the Punjab, Lahore 54590, Pakistan; znawaz@pucit.edu.pk (Z.N.); faisal.bukhari@pucit.edu.pk (F.B.); 4 Punjab Institute of Neurosciences (PINS), Lahore General Hospital, Lahore 54000, Pakistan; ammaranwer@hotmail.com; 5 Departments of Radiology and Pathology, Massachusetts General Hospital, Harvard Medical School, Boston, MA 02129, USA; cheng@nmr.mgh.harvard.edu

**Keywords:** alanine, glioma, high-resolution magic angle spinning nuclear magnetic resonance spectroscopy (HRMAS-NMRS), untargeted metabolomics, machine learning, liquid biopsy

## Abstract

Metabolic alterations play a crucial role in glioma development and progression and can be detected even before the appearance of the fatal phenotype. We have compared the circulating metabolic fingerprints of glioma patients versus healthy controls, for the first time, in a quest to identify a panel of small, dysregulated metabolites with potential to serve as a predictive and/or diagnostic marker in the clinical settings. High-resolution magic angle spinning nuclear magnetic resonance spectroscopy (HRMAS-NMR) was used for untargeted metabolomics and data acquisition followed by a machine learning (ML) approach for the analyses of large metabolic datasets. Cross-validation of ML predicted NMR spectral features was done by statistical methods (Wilcoxon-test) using JMP-pro16 software. Alanine was identified as the most critical metabolite with potential to detect glioma with precision of 1.0, recall of 0.96, and F1 measure of 0.98. The top 10 metabolites identified for glioma detection included alanine, glutamine, valine, methionine, N-acetylaspartate (NAA), γ-aminobutyric acid (GABA), serine, α-glucose, lactate, and arginine. We achieved 100% accuracy for the detection of glioma using ML algorithms, extra tree classifier, and random forest, and 98% accuracy with logistic regression. Classification of glioma in low and high grades was done with 86% accuracy using logistic regression model, and with 83% and 79% accuracy using extra tree classifier and random forest, respectively. The predictive accuracy of our ML model is superior to any of the previously reported algorithms, used in tissue- or liquid biopsy-based metabolic studies. The identified top metabolites can be targeted to develop early diagnostic methods as well as to plan personalized treatment strategies.

## 1. Introduction

Malignant brain tumors such as glioma and glioblastoma multiforme (GBM), arising from the glial cells of the central nervous system (CNS), are among the most lethal forms of human cancers. Their aggressive nature, infiltrating growth, and a two-fold blood–brain and blood–brain–tumor barrier (unlike most other cancers) make them both difficult to diagnose early and challenging to treat [1,2,3]. Therefore, despite surgical resection and advanced multimodal treatment modalities available currently such as biodegradable medicated wafer implants, postoperative concomitant chemo-radiation therapy (Stupp regimens), maintenance adjuvant chemotherapy, Optune^®^—tumor treating electric fields, immuno-chemotherapy—and so on, the recurrence and mortality rates of malignant gliomas are significantly higher than other solid and hematological malignancies; the median overall survival of the patients is <1.2 years [3,4,5,6,7,8].

Although our understanding of the intricate molecular networks and/or their cross-talks that initiate the successive, yet aggressive series of proliferative events in gliomas is very limited, there is increasing evidence suggesting that a complex interplay of chromosomal alterations (gene-gene fusions), genetic aberrations (point mutations), and epigenetic modifications (methylations) contributes to the tumor biogenesis [3,9,10]. Besides this, the underlying concealed perturbations in several finely tuned signaling (such as p53, VEGF, ErbB, RTK, Akt) and metabolic pathways (for instance, choline, central-carbon, and glutamine) also drive and accelerate the metastatic capabilities of gliomas [10,11,12,13]. Several research groups have demonstrated that, in order to perform (i) harvesting of energy and replenishing the ‘nutrient (glucose) sink’ for a continuous, unchecked cellular proliferation; (ii) epithelial-to-mesenchymal transition (EMT); (iii) enhanced cell migration; and iv) increased angiogenesis, the cancer cells establish a preference for glycolytic metabolism—a so-called Warburg effect [13,14,15]. This metabolic reprogramming/shift from the oxidative phosphorylation to glycolytic pathway coupled with concomitant upregulated expression of the solute carrier 2 (SLC2) family of transporters (e.g., glucose transporter GLUT1, GLUT3, GLUT4) is the central hallmark of malignant gliomas [13,15,16]. While on one hand, it exerts a selective pressure for tumor survival in the tumor microenvironment, at the same time, it drastically perturbs the metabolic influx of glucose within the cell cytoplasm and in circulation (more importantly, blood plasma, which is supplying nutrients to the entire body).

Given that the pronounced alterations in metabolic pathways and their corresponding metabolites manifest during the course of ‘tumor initiation-to-progression’ and/or in response to the treatment in different cancers, we have compared the metabolic fingerprints of glioma patients versus healthy controls in a quest to identify a panel of small, dysregulated, circulating metabolites with the potential to serve as a predictive- and/or diagnostic markers in the clinical settings. One of the most advanced and sophisticated techniques, high resolution magic angle spinning nuclear magnetic resonance spectroscopy (HRMAS-NMR), has been used for untargeted metabolomics and data acquisition in conjunction with the machine learning (ML) approach to analyze the large metabolic datasets. HRMAS-NMR is considered to be advantageous over the latest mass spectrometric methods thanks to its high reproducibility without the need for any pre-measurement treatment, robustness, non-destructive nature, minimum sample preparation time, and ability to provide the qualitative as well as the quantitative fingerprints of all measurable metabolites in a single experiment [17,18,19,20]. However, the data obtained from HRMAS-NMR are usually complex; raw NMR signal processing and targeted analysis pipeline often require an expert to reveal the biological insights. We have thus combined the HRMAS-NMR with ML-based data mining algorithms to assist in the traditional chemo-metric analysis. This, to the best of our best knowledge, is the first study that systematically, yet in less time and with minimized human biasness/intervention, assesses the plethora of small circulating metabolites in blood, both qualitatively and quantitatively, and presents a sensitive marker demonstrating the highest glioma-predictive accuracy (precision 1.0; recall 0.96; F1 measure 0.98) ever reported.

## 2. Results

### 2.1. Clinicopathological Characteristics of Study Group

Untargeted metabolic profiling was performed by HRMAS-NMR using plasma samples (*n* = 42) obtained from low-grade (LGG, *n* = 9) and high-grade glioma (HGG, *n* = 17) patients along with healthy controls (*n* = 16). The primary demographics and clinical characteristics of enrolled patients are shown in Table 1. Briefly, 60% of the enrolled patients were males, while 40% were females, with the average age of patients being 38 years. Out of the 93 initially registered LGG and HGG cases, only those patients were included in the study who were confirmed by histopathological analysis of tumor tissue and were graded according to the World Health Organization (WHO) classification of brain tumors. The glioma patients having any metabolic disorder (i.e., diabetes mellitus), hypertension, liver (hepatitis/liver cirrhosis), and/or cardiovascular disease were excluded from the study.

### 2.2. Correlating Circulating Metabolomic Profiles for Glioma Detection

**Figure 1:**
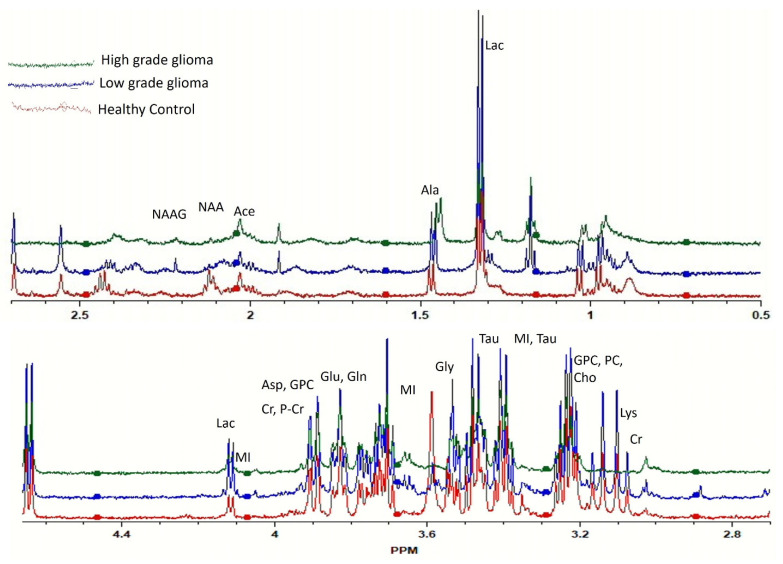
Comparison of high-resolution magic angle spinning nuclear magnetic resonance spectroscopy (HRMAS-MRS) spectra of healthy (red), low-grade glioma (LGG, blue), and high-grade glioma (HGG, green) study subjects. Important peaks are labelled.

The representative HRMAS-NMR spectra obtained from the plasma samples of LGG, HGG, and the healthy control groups are shown as Figure 1 (peaks of important metabolites are labelled). The curve fitting of spectra from 4.66 to 0.50 ppm identified the resonance peaks in 251 regions out of the 417 spectral regions (the remaining 166 regions mostly contained zeros), which were selected for further analysis after applying the feature selection techniques.

**Figure 2:**
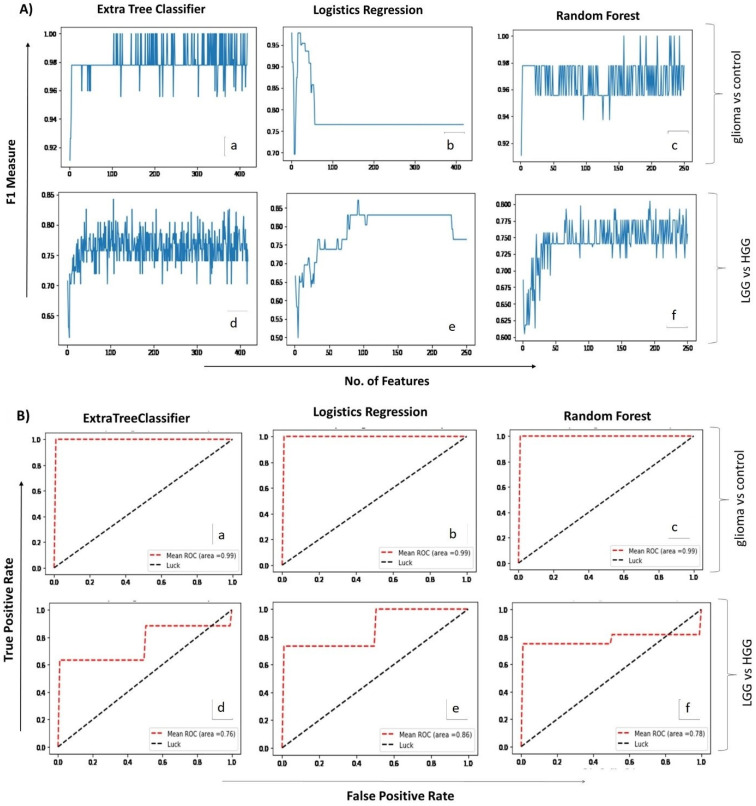
(**A**). Mean score plots displaying the number of features (x-axis) and the corresponding F1 measures (y-axis) obtained for each of the three ML algorithms applied (i.e., extra tree classifier, logistic regression & random forest) for glioma vs control (**a**–**c**) and LGG vs HGG analysis (**d**–**f**), respectively. (**B**). Receiver operating curve (ROC) plots depicting predictive accuracy of the three ML algorithms for glioma vs control (**a**–**c**) and LGG vs HGG (**d**–**f**) groups, respectively. X-axis shows false positive rate while Y-axis indicates true positive rate on a scale of 0 to 1.0.

Analysis of glioma versus control groups (LGG + HGG = 26, healthy control = 16) using the two ML algorithms, extra tree classifier and random forest, identified 104 and 158 spectral regions, respectively, that differentiated the glioma samples from the healthy controls with 100% accuracy (F1-measure: 1.00). Both models gave maximum accuracy, but the number of identified spectral regions was too high to be supportive in routine analyses; therefore, we used the third ML model, i.e., logistic regression (Figure 2A). Interestingly, the predictive accuracy of this model with single spectral region was 98% (F1-measure: 0.98). The aggregate confusion matrix and fivefold cross-validations applied to estimate the prediction error/evaluate the performance of each fitted model revealed only one false negative prediction with logistic regression, whereas none were revealed by extra tree classifier and random forest. The performance of all ML models used for glioma detection and classification is summarized in Table 2.

The logistic regression model was regarded as best as it could differentiate glioma samples from the healthy controls with 98% accuracy using a minimum number of spectral regions (*n* = 1). The spectral regions identified by other two ML models were arranged in descending order of importance and the top 30 were selected for metabolite identification. Major contributing metabolites (*n* = 24) corresponding to these 30 spectral regions were searched from the available literature and databases and are summarized in Table 3. The single spectral region identified by the logistic regression model (1.47) was unveiled to be ‘alanine’ and it was also found to be significantly decreased in glioma cases. Other top dysregulated metabolites for glioma versus control cases included methionine, N-acetylaspartate (NAA), valine, glutamine, γ-aminobutyric acid (GABA), serine, α-glucose, lactate, and arginine.

### 2.3. Correlating Circulating Metabolomic Profiles for Glioma Classification (LGG/HGG)

A second set of ML analyses was performed to identify the spectral regions that may potentially differentiate and classify low- (grade I–II) and high-grade (grade III–IV) gliomas ((total 25 samples in two groups (LGG = 9, HGG = 16)). Logistic regression presented the highest F1-measure (0.86) and identified 92 spectral regions, whereas extra tree classifier and random forest presented 0.83 and 0.79 F1-measures, while identifying 107 and 88 spectral regions, respectively. The top 30 spectral regions contributing towards detection and classification of glioma are listed in Table 3 in descending order of importance. Their corresponding dysregulated metabolites’ search came up with 22 metabolites that included choline, NAA, valine, succinate, GABA, creatine, isoleucine, glutamine, fatty acids, and taurine, among others. The diagnostic ability of each predictive model used was further checked by ROC plots (Figure 2B), which showed high sensitivity of all three models for glioma versus control analyses, with the area under the curve (AUC) being 0.99. For the second set of analyses (classification of glioma in low- and high-grades), the logistic regression model proved to be more sensitive (AUC = 0.86) in comparison with extra tree classifier (AUC = 0.76) and random forest (AUC = 0.78). Additional analysis performed using a separate test dataset of oligodendroglioma patients revealed the usefulness of ML models. Extra tree classifier and random forest detected the presence of tumor by high accuracy (F1-measure = 0.91). Logistics regression detected the tumor by selecting only one feature (i.e., alanine) and scored an F1-measure value of 0.75 (Supplementary Data: Table S2). The overall results point towards the usefulness of ML-assisted circulating metabolite profiling for both the detection and classification of gliomas.

### 2.4. Identification of Statistically Significant Dysregulated Metabolites and Glioma-Associated Pathways

The ML-predicted top 30 spectral regions and corresponding dysregulated metabolites along with their statistical significance (*p*-value) and fold-change value (log2FC), in both the ‘glioma versus control’ and ‘LGG versus HGG’ comparisons, are listed in Table 3. Altogether, statistical analysis identified 98 spectral regions with a *p*-value < 0.05 for glioma versus control groups. Interestingly, except 3.72 and 3.47 ppm regions (the contributing metabolite for both spectral regions is β-glucose), all top 30 ML-predicted spectral regions were found to be statistically significant, adding to the predictive accuracy of ML analyses (Table 3 shaded boxes). For the LGG versus HGG group, however, only 19 ML-predicted spectral regions were regarded as statistically significant (*p* < 0.05).

**Figure 3:**
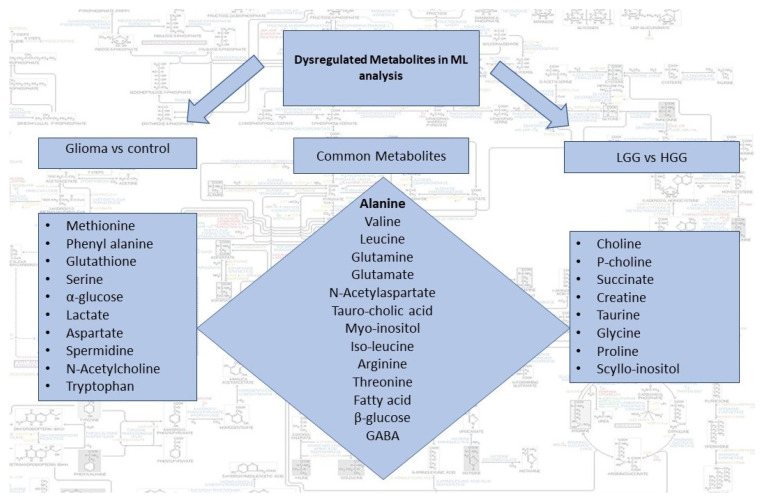
Summary of dysregulated metabolites identified by the two sets of ML analysis i.e., glioma vs control and LGG vs HGG. Common metabolites can potentially be used for both detection and classification of gliomas.

**Figure 4:**
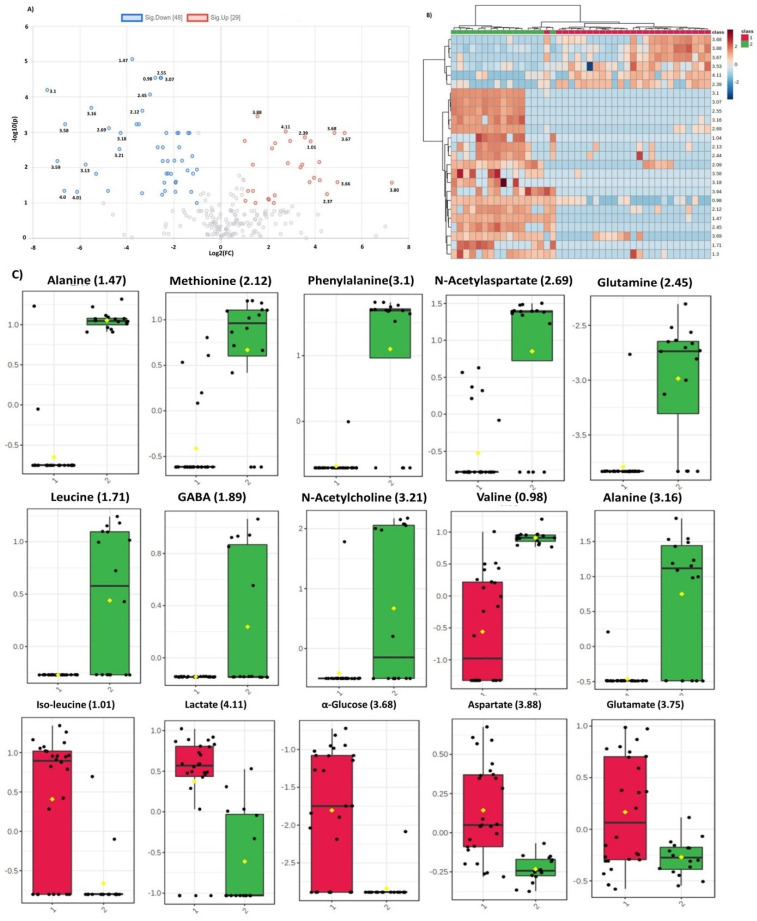
(**A**) Volcano plot representing fold change in the dysregulated metabolites for glioma vs control group. Left and right panels of the plot respectively indicate the down- and upregulated spectral regions. Alanine (1.47) was identified as most significant metabolite with highest value of –log10(p). (**B**). Heatmap of top 30 spectral regions identified for glioma vs control group. Class 1 represents the glioma samples while class 2 the control group. Each colored cell corresponds to normalized intensity value of selected feature. The difference(s) between the two groups can be visualized by change in color. Upregulated and downregulated spectral regions can be seen clearly in two classes. (**C**) Box and Whisker plots of two groups (1=glioma, 2=control) representing mean change in intensity values of the selected metabolites. Notch of the plot indicates the median value while yellow diamond shows mean value of the feature in the group.

Dysregulated metabolites contributing to significant spectral regions for both groups of analysis and common in two comparison sets are summarized in Figure 3. Alanine was the only metabolite detected by all the three ML models for glioma detection. Other important metabolites for glioma detection included methionine, phenylalanine, glutathione, serine, glucose, lactate, aspartate, NAA, and tryptophan. For glioma classification in low and high grades, choline, p-choline, succinate, creatine, taurine, glycine, proline, and scylloinositol were regarded as the key metabolites. The fold-change and mean intensity values for selected dysregulated metabolites, in all three study groups, are presented in Figure 4 in the form of heatmaps, volcano, and box and whisker plots.

**Figure 5:**
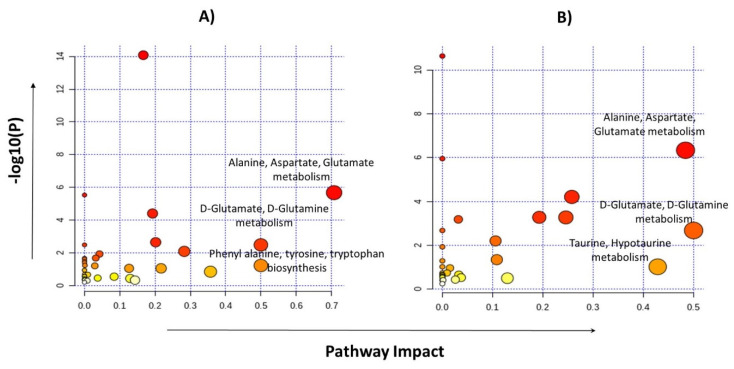
Pathway analysis performed by MetaboAnalyst using metabolites identified for glioma vs control group (**A**) and the LGG vs HGG group (**B**).

Pathway analyses were performed by selecting the 24 metabolites identified for glioma detection and 22 metabolites for glioma classification separately. Major pathways found to be dysregulated in both analysis groups are shown in Table 4. The highest impact generating pathways for glioma detection were found to be associated with alanine/aspartate/glutamate metabolism (impact = 0.7) followed by D-glutamine/D-glutamate metabolism (impact = 0.5) and phenylalanine/tyrosine/tryptophan anabolism (impact = 0.5). Metabolic pathways that created a maximum impact for glioma classification were linked with glutamine/glutamate metabolism (impact = 0.5) and the alanine/aspartate/glutamate metabolism (impact = 0.48), as shown in Figure 5.

## 3. Discussion

Tumor development in the glial cells (the cells that typically modulate the brain microenvironment, homeostasis, and neurochemical balance in the CNS) is a complex process, often accompanied by reprogramming of key metabolic pathways and the rewiring of central carbon metabolism. Several studies have shown that the survival and growth of tumor cells is dependent on prompt availability of nutrients/metabolic fuels, which in turn is linked with activation and/or deactivation of several pathways, especially those of carbohydrate (e.g., glucose) and amino acids (e.g., glutamine, glutamate) metabolism [10,11,15,21,22]. As metabolic alterations in tumor cells have a pronounced impact on the profile of circulating metabolites, these may serve as an attractive target for non-invasive diagnosis of disease and prognosis of therapeutic response, without essentially going through the conventional MRI, CT scans, and invasive tissue biopsy/histopathology analyses. However, how these metabolic perturbations manifest in the context of different malignancies like gliomas (in particular, GBMs) is a formidable challenge for the oncologists and clinicians.

Previously, a targeted analysis of plasma metabolites using mass spectrometric methods identified 18 statistically significant metabolites that could differentiate LGG from the HGG samples [23,24,25]. Another study reported three plasma metabolites that were found to be associated with survival in GBM patients [26,27]. Yet, to the best of our knowledge, no study has reported liquid biopsy-based detection of glioma-associated metabolic signatures combining the untargeted HRMAS-NMR with ML. ML-based data mining algorithms, often conducted in an unsupervised manner, can reduce the human biasness and minimize analysis time, while processing the large datasets obtained from the metabolomics experiments. ML models have strong potential to be used in biomarker research as they can identify the best features contributing to a particular phenotype, classify diseases, and predict possible outcomes while utilizing the prior experiences obtained from the training datasets [28,29].

In the present study, using untargeted HRMAS-NMR spectroscopy and applying the ML linear logistic regression model, we interestingly identified a single metabolic marker “alanine” that could differentiate glioma from non-glioma samples with the highest predictive accuracy ever reported (precision 1.0; recall 0.96; F1 measure 0.98). Alanine is a glucogenic amino acid, which, following enzymatic conversion to pyruvate, enters the metabolic mainstream to provide energy and replenish the nutrient sink for rapidly proliferating tumor cells [30] Interestingly, alanine was the only top-scorer metabolite that was identified as a potential metabolic marker by all three ML models for glioma detection (Table 3; Figure 3 and Figure 4A,C). Other potential candidate metabolites that we identified for liquid biopsy-based detection and/or classification of gliomas include essential, non-essential, and branched-chain amino acids (e.g., Glu, Gln, Met, Leu, Ile, Val, Arg, Thr); neurotransmitters (NAA, GABA); fatty acids; and D-glucose (Figure 3).

Whereas the aggregate confusion matrix of all the ML algorithms (Table 2) classified the 16 HGG cases with 95–100% accuracy, it misclassified over 50% of LGG cases. In particular, logistic regression accurately predicted and classified all HGG samples, but, out of 09 LGG cases, it misclassified 05 as HGG. Extra tree classifier and random forest misclassified 05 and 07 LGG patients as HGG, respectively, and one HGG patient (the same patient in both models) as an LGG case. This LGG misclassification could primarily be the result of the small number of LGG cases (*n* = 9) used to train/test the ML algorithms. However, a deeper appreciation of the clinical record of the LGG subjects revealed that all misclassified LGGs were isocitrate dehydrogenase (IDH) wild-type cases. Studies have shown that tumors with IDH wild-type genes (also called IDH-negative) tend to progress far more aggressively to grade IV GBM and demonstrate poorer prognosis than their IDH mutant counterparts [31]. We speculated that, by analyzing the spatial distribution of metabolic changes, within the brain tissue and/or circulation using ML-assisted HRMAS-NMRS, one may predict the probability of LGG transition to HGG; however, further studies are warranted to validate the hypothesis.

Altered glutamate/glutamine metabolism is the hallmark of several cancers and, here, we report a significant decrease in plasma glutamine concentration in glioma samples. Glutamate/glutamine cycle in the brain is highly regulated; glutamate is a central amino acid required for neurotransmission, it acts as synaptic excitatory neurotransmitter, and accounts for more than 80% synapses in the brain [15,32]. After neurotransmission, glutamate in synaptic cleft is taken up by the astrocytes, metabolized to glutamine by an enzyme glutamine synthetase, and transported to neurons where it converts back to glutamate, thus completing the glutamine/glutamate cycle [32,33]. Compared with normal cells, the cancer cells are known to metabolize glutamine at higher levels to meet the ATP demands for biosynthesis of required proteins, lipids, and nucleic acids [15,34,35]. Glutamate homeostasis is found to be dysregulated in brain tumors, especially gliomas, and there are studies that report a higher concentration of glutamine in the tumor tissues [36,37]. In particular, the glial cells with IDH mutations have been identified for taking up high levels of glutamine to produce higher levels of 2-hydroxyglutarate [31,37,38]. This increased uptake of glutamine by glial cells may be responsible for lowering its levels in plasma (Figure 4A–C), hence its potential role as an attractive, non-invasive biomarker for detection and/or classification of gliomas.

Branched-chain amino acids (leucine, isoleucine, and valine) are utilized by the tumor cells for de novo synthesis of glutamate, which is important to fulfil the increased demand for glutamate [39,40]. Arginine, a semi-essential amino acid, was also found to be dysregulated in both comparisons, thus significantly contributing to glioma detection. Methionine is a vital amino acid; many different cancer cells have been found to be dependent on it for their growth. An increased requirement for exogenous methionine during cancer development is analogous to the high demands of cancer cells for glucose [41]. A study has reported it to be essential for colony formation and survival of glioma cells [42]. Methionine levels in the present study were found to be significantly decreased in the plasma of glioma patients, which indicates increased expense of this amino acid in the tumor microenvironment (Figure 4).

Lactate is another important metabolite detectable in brain tumors or areas of ischemic injury because of anaerobic glycolysis. High-grade tumors of adults show more prominent lactate peak as compared with low-grade tumors, while all pediatric brain tumors exhibit high lactate levels. Creatine (Cr) along with phosphocreatine is used normally in energy metabolism. Its decreased levels in tumor cells are likely to be the result of its consumption to replenishing energy under conditions when oxidative phosphorylation and anaerobic glycolysis fail to meet the high energy demands of progressive tumors. Lipids are associated with necrosis and are present in higher amounts in glioblastoma, lymphoma, abscess, or other areas of destruction of myelinated white matter and, subsequently, decrease in circulation [43]. Our results substantiate the previous studies signifying the role of dysregulated plasma metabolic profiling in detection and/or classification of gliomas [23,41,42,43,44].

## 4. Materials and Methods

### 4.1. Study Population and Sample Collection

The study population is comprised of 42 subjects that include both low-grade (LGG; *n* = 9) and high-grade glioma patients (HGG; *n* = 17) along with 16 healthy controls. The study design was duly approved by the Advanced Studies and Research Board (ASRB; 2402-3/2018), University of the Punjab, Lahore. LGG and HGG patients from Punjab Institute of Neurosciences (PINS) (diagnosed by expert oncologists using magnetic resonance imaging (MRI) or computerized tomography (CT) scan, undergoing surgical resection of the tumor) were recruited during March 2018–2019. Clinical and demographic data of the enrolled patients were obtained from medical record of the hospital. Patients’ recruitment and sample collection followed the principals of the Declaration of Helsinki for research involving human beings. Written informed consent from all the study subjects was obtained prior to drawing their blood sample.

### 4.2. Ex Vivo HRMAS-NMR

Peripheral blood (3 cc) from each patient (fasting state) was collected in Li-heparin tubes, centrifuged (300× *g*, 10 min) to prepare plasma within an hour of collection, and preserved in sterile tubes at −80 °C, as 200 µL aliquots, until further analyses.

The HRMAS-NMR analyses were performed on Bruker AVANCE 600 MHz spectrometer (Billerica, MA, USA) equipped with a triple nucleus (1 H, 13 C, 31 P) HRMAS probes. The spectra were acquired by adding 10 µL plasma sample in a 4 mm zirconia rotor with 12 μL Kel-F inserts; 2 µL D2O (Sigma Aldrich, St. Louis, MO, USA) with reference trimethylsilylpropanoic acid (TSP) was added for field locking. For all measurements, 4 °C temperature, 3600 ± 2 Hz spin-rate (to keep the rotation sidebands out of the acquisition window), and a rotor-synchronized Carr-Purcell Meibom-Gill (CPMG) pulse sequence (to function as a T2-filter) were used. To reduce the metabolites’ variation, each spectrum was recorded at 4 °C with and without continuous wave water suppression, with spectrometer frequency centered on water resonance [45].

### 4.3. Pre-Processing of the Spectral Data

Acorn NMR-Nuts (Livermore, CA, USA) software was used to process the acquired HRMAS-NMR spectra. Free induction decay (FID) file of each spectrum was subjected to a successive chain of commands to perform baseline correction (bc), exponential multiplication (em, 0.5 Hz line broadening), zero filling (zf), rotation control (rc = 68), Fourier transformation (ft), spectra reversing (sr), and automatic phasing (ap). All spectra were processed and aligned against TSP at 0 ppm that placed creatine (Cre) methyl resonance at 3.026 ppm. Peak intensities between the spectral regions 4.66 and 0.5 ppm were curve fitted to the accuracy of 0.01 ppm, and normalized to the relative spectral intensity (Re*l*_*Int*) according to the formula below.

(%Rel_Intm,s)=(Exp_Intm,s)∗100/∑i=1251(Exp_Inti,s)

where (*Exp_Int_m,s_*) represents the experimental intensity value for spectral regions *m* (*m* = 1, 2, 3, …251) and samples *s* (*s* = 1, 2, 3, ……42), and $\sum_{i=1}^{251}\left(Exp\_Int_{i,s}\right)$ represents the sum of intensity values measured for all selected 251 spectral regions, for each of the 42 samples.

### 4.4. ML-Assisted Data Analysis

Both linear (logistic regression) and non-linear (random forest, extra tree classifier) ML classification methods were used for the accurate prediction of the ‘spectral regions’ essentially linked with the glioma and non-glioma groups. The dataset was normalized by applying Z-score normalization followed by log scaling. Feature selection technique, involving analysis of variance (ANOVA), feature importance, and recursive feature elimination methods, was implemented to reduce the dimensionality of large dataset and to select the best spectral regions associated with the disease. The HRMAS data initially contained 417 spectral regions for analysis. However, after initial selection, only 251 regions were found to have a comparable numeric value; features containing more zero and/or missing values were all excluded from the dataset. Further ML analyses were performed with only those spectral regions that have had the highest impact on the response variable.

To ‘train’ and ‘test’ the dataset for the best possible predictions of glioma versus non-glioma controls, a comprehensive ML approach was followed wherein a wide choice of hyper-parameters was used for each model using GridSearchCV. A fivefold cross-validation was performed at each step to avoid overfitting problem in the dataset. In particular, the data were divided into five equal parts, 80% of which was used to train the ML model, while the remaining 20% was used to test the model function, reducing the chances of overfitting thenceforth. Different evaluation metrics, such as accuracy, recall, precision, and F1-measure (chosen for its best presentations in cases of the multiclass problems of imbalanced data), were computed for each model using the formulas:

Precision = True positive/True positive + False positive
Recall = True positive/True positive + False negative
F1-measure= 2 × Precision × Recall/Precision + Recall

The analyses were repeated for LGG versus HGG cases, and the highest value of F1 measure was recorded by looping through every model, feature, and hyper-parameters. The diagnostic potential of each predictive model was determined by the receiver operating characteristic curve (ROC) plots. A separate test dataset comprising six plasma samples from oligodendroglioma patients was also included to evaluate the tumor versus non-tumor predictive accuracy of ML models.

### 4.5. Statistical Analysis

Statistical analysis, performed using JMP-pro16 software, were used to further validate the spectral regions identified by ML algorithms. Shapiro–Wilk non-parametric test was applied to check the normality of the given dataset, while the Wilcoxon test to find out the statistically significant spectral regions for glioma versus control and LGG versus HGG groups, separately. A comprehensive platform MetaboAnalyst 5.0 (available at: https://www.metaboanalyst.ca/MetaboAnalyst/home.xhtml) (accessed on 13 July 2021) was used to calculate the spectral fold change (FC; mean value of change) between the two groups.

### 4.6. Metabolite’s Identification and Pathway Analysis

Literature search and publicly available databases such as Human Metabolome Database (HMDB), Biological Magnetic Resonance Databank (BMRB), and MetaboMiner and MetaboHunter, were used for the identification/assignment of metabolites to the corresponding top 30 spectral regions, predicted by the ML models. Further, the metabolic pathways dysregulated during the tumor progression were identified using MetaboAnalyst 5.0.

Metabolites of interest, quantified by selected distinctive unbiased NMR signals and identified to be important after ML-based analyses, were used as the input matrix. The pathway impact was calculated as the sum of the important measures of the matched metabolites normalized by the sum of the important measures of all metabolites in each pathway.

## 5. Conclusions

In conclusion, the advanced HRMAS-NMR in conjunction with ML algorithms may serve as a pathway to predict the glioma risk, occurrence, and/or grading easily, reliably, and accurately. The dysregulated circulating metabolite, alanine, may be tested non-invasively using routine spectrophotometric methods in the future, enabling real-time monitoring or objective evaluation of tumor progression, treatment response, and more individualized prognostication. The double-blind validation studies with a larger number of plasma samples from patients at different stages and grades of glioma are, however, necessary to cross-verify the reliability and accuracy of ML models and add support to our findings.

## Figures and Tables

**Table 1:** Demographics and clinical characteristics of study subjects.

Characteristics	LGG (grade I–II)	HGG (grade III–IV)	Healthy Control	Total
No. of Subjects (n)	4 + 5 = 9	1 + 16 = 17	16	42
Mean Age (Years)	33 ± 17	43 ± 16	34 ± 13	38 ± 16
Gender				
Male	07	11	07	25
Female	02	06	09	17
Headache				
Yes	05	13	0	18
No	04	04	16	24
Epileptic Seizures				
Yes	03	07	0	10
No	06	10	16	32
Gait/Balance Changes				
Yes	05	13	0	18
No	04	04	16	24
Neurologic Deficits				
Yes	07	15	0	22
No	02	02	16	20
Reduced Vision				
Yes	03	08	0	11
No	06	09	16	31
Cancer History				
Yes	01	03	0	04
No	08	14	16	38

**Table 2:** Performance and confusion matrices of machine learning (ML) algorithms applied for the detection and classification of glioma in the study subjects.

Sample Type	Algorithm Applied	Confusion Matrices of ML Algorithms ^1^	No. of Features Identified ^2^	Group	Precision	Recall	F1-Measure
Glioma vs. Control (*n* = 42)	Extra Tree Classifier	[16 0]	104	Control	1.00	1.00	1.00
		[0 26]		Tumor	1.00	1.00	1.00
	Logistic Regression	[16 0]	01	Control	0.94	1.00	0.97
		[1 25]		Tumor	1.00	0.96	0.98
	Random Forest	[16 0]	158	Control	1.00	1.00	1.00
		[0 26]		Tumor	1.00	1.00	1.00
LGG vs. HGG (*n* = 25)	Extra Tree Classifier	[4 5]	107	LGG	0.80	0.44	0.57
		[1 15]		HGG	0.75	0.94	0.83
	Logistic Regression	[4 5]	92	LGG	1.00	0.44	0.62
		[0 16]		HGG	0.76	1.00	0.86
	Random Forest	[2 7]	88	LGG	0.67	0.22	0.33
		[1 15]		HGG	0.68	0.94	0.79

^1^ Key used: [true negative false positive]; [false negative true positive]; ^2^ details of features/spectral regions identified in each case are provided in the Supplementary Material as Table S1.

**Table 3:** Top 30 spectral regions and their contributing metabolites identified by both sets of ML analysis given in descending order of importance along with their statistical analysis.

Group 1: Glioma vs Control				
ML Analysis			Statistical Validation	
Feature	Importance	Corresponding metabolite	*p*-value	log2(FC)
***Model: Logistics Regression***				
1.47	1.0	Alanine	<0.0001	–3.7744
***Model: Extra Tree Classifier***				
2.55	0.0642	β-Alanine	<0.0001	−2.5717
2.12	0.0524	Methionine	<0.0001	−3.3458
3.1	0.0491	Phenylalanine	<0.0001	−7.3967
3.07	0.0415	X	<0.0001	−2.5445
2.69	0.0405	NAA	<0.0001	−4.7668
1.47	0.0383	Alanine	<0.0001	−3.7744
0.98	0.0299	Valine	<0.0001	−2.7959
1.71	0.028	Leucine	<0.0001	−3.5926
3.13	0.025	Glutathione	0.0013	−5.7592
2.45	0.0222	Glutamine	<0.0001	−3.0169
1.88	0.022	GABA	0.0028	−1.1886
3.95	0.0204	Serine	0.0003	−2.4757
3.16	0.0182	Alanine	<0.0001	−5.5148
3.69	0.0179	α-glucose	<0.0001	−1.3382
1.44	0.0175	Deoxycholic acid	<0.0001	10.114
3.18	0.0168	Taurocholic acid	<0.0001	−4.2704
4.11	0.0161	Lactate	<0.0001	2.7555
1.72	0.0157	Arginine	0.0003	−2.6918
3.58	0.0149	Threonine	<0.0001	−6.6301
2.35	0.0148	Glutamate	0.0022	−1.4198
3.94	0.0143	Serine	<0.0001	−2.3249
3.14	0.014	Spermidine	0.0119	−4.8047
3.88	0.014	Aspartic acid	<0.0001	1.5565
2.09	0.0133	Glutamate	<0.0001	−1.859
3.21	0.0126	N-Acetylcholine	0.0004	−4.3221
1.04	0.0126	Valine	<0.0001	−1.8092
1.01	0.0124	Isoleucine	0.0002	3.8182
1.89	0.0121	GABA	0.001	−2.2659
3.68	0.0118	α-glucose	<0.0001	4.8319
1.99	0.0117	Isoleucine	0.0006	−1.2174
***Model: Random Forest***				
2.12	0.0491	Methionine	<0.0001	−3.3458
0.98	0.0488	Valine	<0.0001	−2.7959
3.16	0.0438	Alanine	<0.0001	−5.5148
4.11	0.0344	Lactate	<0.0001	2.7555
1.47	0.0316	Alanine	<0.0001	−3.7744
3.53	0.03	Myoinositol	0.0003	1.0134
1.45	0.025	Isoleucine	0.0002	2.9475
1.44	0.0209	Deoxycholic acid	<0.0001	10.114
1.99	0.0195	Isoleucine	0.0006	−1.2174
2.69	0.019	NAA	<0.0001	−4.7668
2.32	0.0172	Glutamate	0.0003	2.1726
2.13	0.0172	Glutamine	<0.0001	−3.4879
2.45	0.0165	Glutamine	<0.0001	−3.0169
3.66	0.0157	Isoleucine	0.0050	4.9598
3.72	0.015	β-glucose	0.3716	NA
1.3	0.0146	Fatty acids	<0.0001	−1.3971
3.94	0.0144	Serine	<0.0001	−2.3249
3.69	0.0142	α-glucose	<0.0001	−1.3382
1.01	0.0142	Isoleucine	0.0002	3.8182
0.92	0.0139	Isoleucine	0.0002	2.3071
2.09	0.0133	Glutamate	<0.0001	−1.859
3.88	0.0117	Aspartate	<0.0001	1.5565
3.59	0.0114	L-Valine	0.001‘	−6.9614
Age	0.0107	NA	NA	NA
1.17	0.0105	X	0.006	−2.41
1.88	0.0105	GABA	0.0028	−1.1886
3.46	0.0097	β-glucose	0.0178	NA
3.22	0.0093	Arginine	0.0191	NA
3.58	0.0091	Threonine	<0.0001	−6.6301
3.47	0.0084	β-glucose	0.7007	NA
**Group 2: LGG vs HGG**				
**ML Analysis**			**Statistical Validation**	
**Feature**	**Importance**	**Corresponding metabolite**	***p*-value**	**log2(FC)**
***Model: Logistics Regression***				
3.51	0.2059	Choline	0.030	NA
2.01	0.1116	NAA	0.041	1.239
3.2	0.0719	P-Choline	0.127	NA
1.02	0.0531	Valine	0.040	NA
3.48	0.0523	β-Glucose	0.040	NA
2.39	0.0442	Succinate/Malate	0.040	−1.1293
1.68	0.0441	L-Arginine	0.227	1.678
1.82	0.0435	X	0.015	1.4804
2.4	0.0416	Succinate	0.092	NA
2.31	0.0386	X	0.207	−1.647
3.5	0.0324	NAA	0.133	−2.3824
2.3	0.0311	GABA	0.133	−3.0367
1.84	0.026	X	0.054	1.6947
3.53	0.026	Myoinositol	0.871	NA
1.26	0.0236	Isoleucine	0.064	−2.4499
1.99	0.0171	Isoleucine	0.039	NA
2.45	0.0156	L-Glutamine	0.195	NA
0.88	0.015	Fatty acid	0.239	−2.0748
3.91	0.0149	Creatine	0.054	1.2722
1.21	0.0125	X	0.054	3.6568
2.25	0.0122	Fatty acid	0.206	2.6199
0.9	0.012	Fatty acid	0.041	1.6497
3.26	0.0028	Taurine	0.009	1.1339
1.83	0	X	0.388	−1.791
1.63	0	X	0.195	NA
1.86	0	GABA	0.206	1.9942
1.97	0	Isoleucine	0.182	1.679
1.47	0	Alanine	0.640	3.2255
1.87	0	GABA	0.195	NA
1.88	0	GABA	0.195	NA
***Model: Extra Tree Classifier***				
3.6	0.0637	Valine	0.015	1.5337
1.82	0.0352	X	0.015	1.4804
2.5	0.0265	NAA	0.015	1.3842
1.4	0.0254	X	0.071	1.5535
2.4	0.0254	Succinate	0.092	1.1227
1.97	0.0247	Isoleucine	0.182	1.679
2.04	0.0244	Glutamate	0.030	1.4984
0.99	0.0237	Isoleucine	0.104	1.2242
2.53	0.0232	X	0.015	2.0478
3.26	0.0232	Taurine	0.009	1.1339
2.08	0.0211	Glutamate	0.036	NA
3.59	0.0207	Threonine	0.053	NA
0.91	0.0186	Fatty acids	0.011	1.6453
0.98	0.017	Valine	0.249	1.7767
1.99	0.0167	Isoleucine	0.039	1.429
0.9	0.0167	Fatty acids	0.041	1.6497
3.33	0.0164	Scyllo inositol	0.015	1.5207
2.43	0.0162	Glutamine	0.222	NA
1.22	0.016	X	0.103	NA
3.91	0.0159	Creatine	0.054	1.2722
2.72	0.0154	NAA	0.136	2.2715
2.84	0.0149	X	0.054	1.2673
3.51	0.0144	Choline	0.030	
0.85	0.0143	Tauro-cholicacid	0.726	−1.0208
1.68	0.0137	Leucine	0.227	1.678
3.54	0.0131	Glycine	0.050	1.1865
1.36	0.0127	Fatty acids	0.519	NA
3.42	0.0124	Taurine/Proline	0.031	NA
1.05	0.0123	Valine	0.097	1.5722
2.32	0.0119	Glutamate	0.026	NA
***Model: Random Forest***				
3.6	0.0637	Valine	0.015	1.5337
1.82	0.0352	X	0.015	1.4804
2.5	0.0265	NAA	0.015	1.3842
1.4	0.0254	X	0.071	1.5535
2.4	0.0254	Succinate	0.092	1.1227
1.97	0.0247	Isoleucine	0.182	1.679
2.04	0.0244	Glutamate	0.030	1.4984
0.99	0.0237	Isoleucine	0.104	1.2242
2.53	0.0232	X	0.015	2.0478
3.26	0.0232	Taurine	0.009	1.1339
2.08	0.0211	Glutamate	0.036	NA
3.59	0.0207	Threonine	0.053	NA
0.91	0.0186	Fatty acids	0.011	1.6453
0.98	0.017	Valine	0.249	1.7767
1.99	0.0167	Isoleucine	0.039	1.429
0.9	0.0167	Fatty acids	0.041	NA
3.33	0.0164	scyllo inositol	0.015	1.5207
2.43	0.0162	Glutamine	0.222	NA
1.22	0.016	X	0.103	NA
3.91	0.0159	Creatine	0.054	1.2722
2.72	0.0154	NAAG	0.136	2.2715
2.84	0.0149	X	0.054	1.2673
3.51	0.0144	Choline	0.030	NA
0.85	0.0143	Tauro-cholicacid	0.726	−1.0208
1.68	0.0137	Leucine	0.227	1.678
3.54	0.0131	Glycine	0.050	1.1865
1.36	0.0127	Fatty acids	0.519	NA
3.42	0.0124	Taurine/Proline	0.031	NA
1.05	0.0123	Valine	0.097	1.5722
2.32	0.0119	Glutamate	0.026	NA

Key used: NAA = N-Acetylaspartate; X = Unknown metabolite; NA = Not available.

**Table 4:** List of metabolic pathways dysregulated for two comparison sets.

Sr.#	Pathway	Glioma vs. Control				LGG vs. HGG			
		Hits	RawP	FDR	Impact	Hits	RawP	FDR	Impact
1	Alanine, aspartate, and glutamate metabolism	06//28	2.13 × 10^−6^	8.34 × 10^−5^	0.70754	06//28	4.57 × 10^−7^	1.92 × 10^−5^	0.48398
2	D-Glutamine and D-glutamate metabolism	2//6	0.00332	0.03984	0.5	2//6	0.00207	0.01937	0.5
3	Arginine biosynthesis	4//14	4.00 × 10^−5^	0.00084	0.19289	3//14	0.00052	0.00739	0.19289
4	Glutathione metabolism	3//28	0.00829	0.08708	0.28281	2//28	0.04453	1	0.12939
5	Aminoacyl-tRNA biosynthesis	13//48	8.36 × 10^−15^	7.02 × 10^−13^	0.16667	10//48	2.30 × 10^−11^	1.93 × 10^−9^	0
6	Glycine, serine, and threonine metabolism	2//33	0.09068	0.40088	0.21707	4//33	0.00053	0.00739	0.24577
7	Arginine and proline metabolism	4//38	0.0023	0.0386	0.20172	5//38	6.13 × 10^−5^	0.00129	0.25763
8	Phenylalanine, tyrosine, and tryptophan biosynthesis	1//4	0.06057	0.31497	0.5	NA			
9	Tryptophan metabolism	1//41	0.47705	1	0.14305	NA			
10	Phenylalanine metabolism	1//10	0.14488	0.55316	0.35714	NA			

## Data Availability

The data that support the findings of this study are available from the corresponding author upon reasonable request.

## Supplementary Materials

The following are available online at https://www.mdpi.com/article/10.3390/metabo11080507/s1, Table S1: List of important spectral regions identified by two sets of ML analysis, Table S2: Results of test dataset (oligodedroglioma vs control) analysis using ML models.
