# Supplementary material for: Dysregulated Alanine as a Potential Predictive Marker of Glioma—An Insight from Untargeted HRMAS-NMR and Machine Learning Data
Source: Metabolites. 2021 Aug 1;11(8):507. doi: 10.3390/metabo11080507 (PMC8402070; doi:10.3390/metabo11080507)
Supplement: Supplementary file 1 [file metabolites-11-00507-s001.zip › metabolites-1260040-SI.pdf]

Table S1. List of important spectral regions identified by two sets of ML analysis

Important spectral regions identified for First Set of Analysis (Glioma vs Control)								
**Logistics Regression (n=1)**	**Extra Tree Classifier (n=104)**			**Random Forest (n=158)**				
1.47	2.55	2.32	3.75	2.12	1.87	2.26	3.21	2.7
	2.12	Age	3.22	0.98	2.1	3.64	3.34	1.46
	3.1	3.53	0.93	3.16	1.16	1.32	2.08	1.26
	3.07	2.38	4.05	4.11	3.33	4.09	4.17	3.39
	2.69	1.91	3.27	1.47	0.96	3.25	2.23	3.02
	1.47	3	4.12	3.53	2.11	1.33	1.15	1.64
	0.98	2.13	2.24	1.45	3.13	0.95	3.37	1.68
	1.71	3.52	0.96	1.44	3.54	0.93	3.62	0.84
	3.13	3.67	1.26	1.99	2.42	2	2.02	
	2.45	2.51	0.88	2.69	1.91	3.92	1.22	
	1.88	3.78	3.35	2.32	3.26	1.98	0.88	
	3.95	2.64	2.06	2.13	4.13	3.42	3.76	
	3.16	2.34	2.11	2.45	3.86	3.78	3.4	
	3.69	3.8	4.63	3.66	1.18	1	1.85	
	1.44	3.91	1.48	3.72	1.03	3.48	4.22	
	3.18	3.33	2.22	1.3	3.55	3.49	1.27	
	4.11	3.92	3.46	3.94	0.87	3.99	1.29	
	1.72	2.21	3.66	3.69	1.31	3.93	2.53	
	3.58	2.1	3.51	1.01	2.41	3.44	2.06	
	2.35	1.87	2.05	0.92	2.35	2.24	1.19	
	3.94	3.73	3.63	2.09	3.83	3.17	1.96	
	3.14	3.49	4.09	3.88	3.73	2.34	3.7	
	3.88	3.83	1.29	3.59	0.91	4.01	4.05	
	2.09	3.59	3.76	Age	3.71	2.43	1.7	
	3.21	3.44	1.15	1.17	3.35	3.91	2.67	
	1.04	3.37	4.23	1.88	3.68	4.65	2.9	
	1.01	3.65	3.41	3.46	3.9	2.04	2.5	
	1.89	3.86	3.97	3.22	3.31	2.05	3.56	
	3.68	1.96		3.58	1.69	3.01	1.25	
	1.99	2.26		3.47	2.37	3.23	1.21	
	3.54	4.13		2.03	3.75	3.82	3.74	
	2.40	1.33		3.8	3.81	0.97	2.31	
	3.96	4.65		3.77	3.03	1.28	0.94	
	2.38	2.03		2.44	3.51	0.99	2.22	
	1.45	3.23		3.1	4.12	3.84	3.41	
	0.92	0.91		3.67	3.65	2.07	3.08	

	0.95	3.4		3.52	3.45	2.33	0.89	
	2.42	2.31		2.40	2.21	3	3.2	
**Important spectral regions Identified for Second Set of Analysis (LGG vs HGG)**								
**Logistics Regression (n=92)**			**Extra Tree Classifier (n=107)**			**Random Forest (n=88)**		
3.51	0.95	2.14	3.6	0.88	3.43	3.6	0.88	3.43
2.01	0.96	2.26	1.82	1.02	3.5	1.82	1.02	3.5
3.2	0.97	4.22	2.5	0.95	1.35	2.5	0.95	1.35
1.02	0.98	2.32	1.4	2.01	3.85	1.4	2.01	3.85
3.48	1.3	2.35	2.4	3.03	3.22	2.4	3.03	3.22
2.39	1.36	2.42	1.97	3.25	1.47	1.97	3.25	1.47
1.68	1.05	2.43	2.04	2.19	2.1	2.04	2.19	2.1
1.82	1.12	2.44	0.99	3.49	4.22	0.99	3.49	4.22
2.4	1.14	2.47	2.53	3.56	2.26	2.53	3.56	2.26
2.31	2.04	2.5	3.26	1.84	1.88	3.26	1.84	1.88
3.5	1.27	2.53	2.08	2.11	0.97	2.08	2.11	0.97
2.3	1.29	2.67	3.59	2.22	2.29	3.59	2.22	2.29
1.84	1.3	2.72	0.91	2.47	1.86	0.91	2.47	
3.53	1.22	2.84	0.98	3.44	3.08	0.98	3.44	
1.26	2.27	0.84	1.99	3.4	2.9	1.99	3.4	
1.99	2.08		0.9	2.35	1.27	0.9	2.35	
2.45	3.54		3.33	1.91	3.87	3.33	1.91	
0.88	3.22		2.43	1.26	1.95	2.43	1.26	
3.91	3.32		1.22	2.99	3.1	1.22	2.99	
1.21	3.33		3.91	3.48	2.44	3.91	3.48	
2.25	3.4		2.72	0.92	1.63	2.72	0.92	
0.9	3.42		2.84	1.29	2.14	2.84	1.29	
3.26	3.43		3.51	0.96	2.2	3.51	0.96	
1.83	3.44		0.85	1.18	3.92	0.85	1.18	
1.63	3.57		1.68	3.57	3.32	1.68	3.57	
1.86	2.1		3.54	1.12	2.45	3.54	1.12	
1.97	3.6		1.36	1.83	4.32	1.36	1.83	
1.47	3.73		3.42	2.31	2.27	3.42	2.31	
1.87	3.85		1.05	1.03	3.94	1.05	1.03	
1.88	3.87		2.32	1.14	1.62	2.32	1.14	
1.95	3.92		1.87	2.25	4.17	1.87	2.25	
1.62	3.94		3.81	0.86		3.81	0.86	
4.32	4.17		3.2	2.88		3.2	2.88	
1.4	3.1		2.42	2.3		2.42	2.3	
0.99	3.08		2.67	3.73		2.67	3.73	
0.85	2.9		1.21	1.3		1.21	1.3	
0.91	2.88		0.84	3.23		0.84	3.23	

0.92	2.11		2.39	3.53		2.39	3.53	

Table S2. Results of test dataset analysis using ML models

Sample type	Algorithm applied	Confusion matrices of ML algorithms[1]	No. of features	Group	Precision	Recall	F1-measure
Oligodendroglioma vs Control (n=6)	Extra Tree Classifier	[0 0]<br>[1 6]	104	Tumor	1.00	0.83	0.91
	Logistic Regression	[0 0]<br>[4 6]	01	Tumor	1.00	0.60	0.75
	Random Forest	[0 0]<br>[1 6]	158	Tumor	1.00	0.83	0.91

[1]key used: [True negative False positive]
[False negative True positive]

[2]Details of features/spectral regions used in each test analysis is provided in supplementary material as Table S1.
